# Supplementary material for: Comparative Metabolome and Transcriptome Analysis of Anthocyanin Biosynthesis in White and Pink Petals of Cotton (Gossypium hirsutum L.)
Source: Int J Mol Sci. 2022 Sep 4;23(17):10137. doi: 10.3390/ijms231710137 (PMC9456042; doi:10.3390/ijms231710137)
Supplement: Supplementary file 1 [file ijms-23-10137-s001.zip › Supplementary figure.pdf]

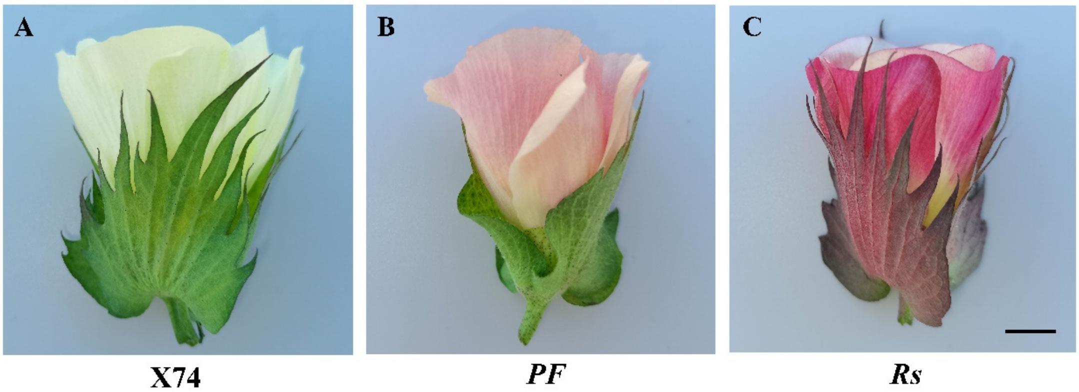


**Figure S1.** Comparison of petal color between **(A)** green leaf cotton XinLuZao 74 (X74), **(B)** *Pink Flower* mutant cultivars (*PF*) and **(C)** sub-red leaf cotton cultivars (*Rs*) grown in natural light. Scale bar: 1 cm.

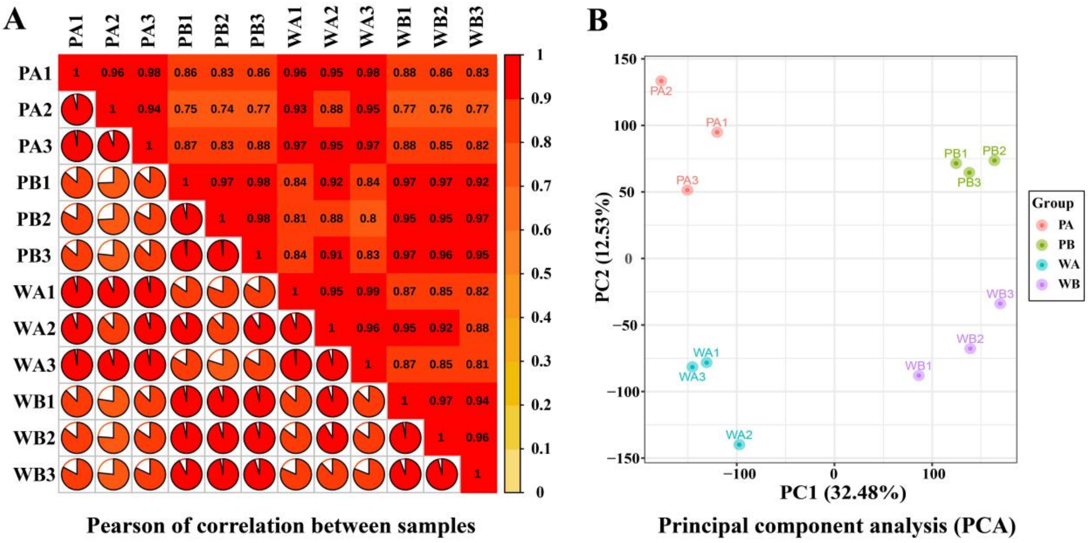


**Figure S2.** Pearson correlation **(A)** and principal component analysis **(B)** between samples. PA, PB, WA and WB represent the spot region and non-spot region of pink and white petals, respectively. PA1/PA2/PA3, PB1/PB2/PB3, WA1/WA2/WA3, and WB1/WB2/WB3 represents three biological replicates for each sample, respectively.

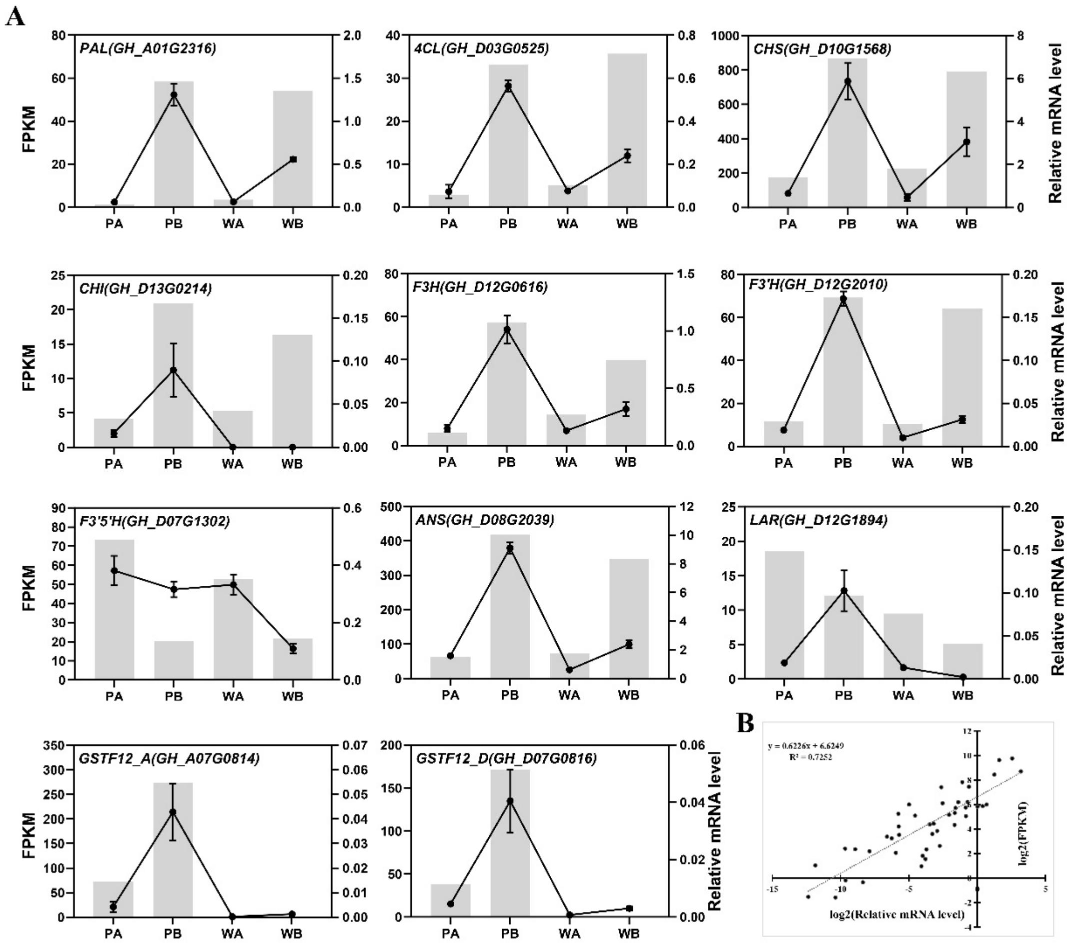


**Figure S3.** Expression analysis of 11 DEGs related to anthocyanin biosynthesis in different samples. **(A)** Transcript levels and qRT-PCR results of 11 selected genes from RNA-sequencing. The left y-axis shows the corresponding RNA-Seq expression data (FPKM) and the right y-axis indicates the relative gene expression levels analyzed by qRT-PCR. Each value in the histogram represents the mean ± standard error. **(B)** The scatter plot of 11 selected genes based on the log2 of the gene expression ratios from qRT-PCR and RNA-seq results indicates the correlation between them. PA, PB, WA and WB represent the spot region and non-spot region of pink and white petals, respectively.

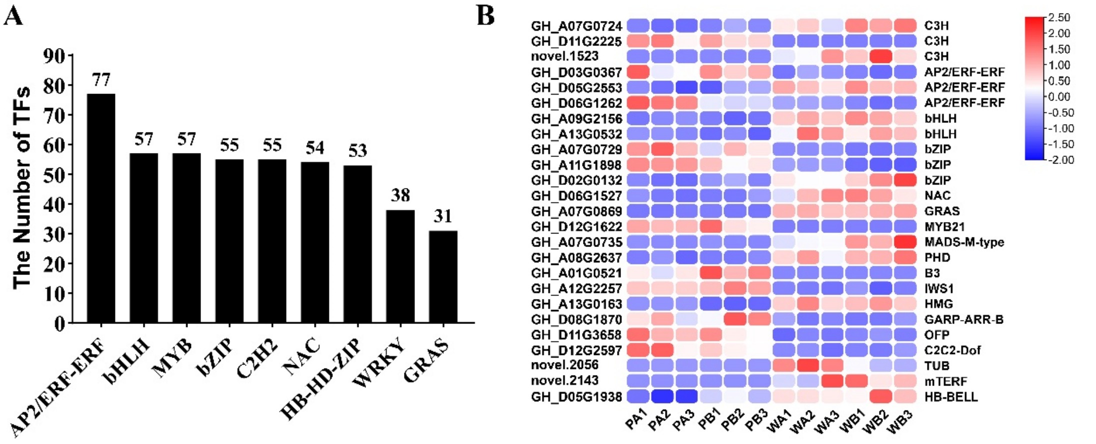


**Figure S4.** Analysis of transcription factors in DEGs. **(A)** The top 9 transcription factors in DEGs. **(B)** The heatmap analysis of 25 transcription factors highly correlated (|PCC| ⩾ 0.9) with Cyanidin-3-O-glucoside. PCC: Pearson's Correlation Coefficient.
