# Supplementary material for: Comparative Metabolome and Transcriptome Analysis of Anthocyanin Biosynthesis in White and Pink Petals of Cotton (Gossypium hirsutum L.)
Source: Int J Mol Sci. 2022 Sep 4;23(17):10137. doi: 10.3390/ijms231710137 (PMC9456042; doi:10.3390/ijms231710137)
Supplement: Supplementary file 1 [file ijms-23-10137-s001.zip › Table S1.docx]

**Table S1.** The primers sequences used in this article

| Q_PAL_F | AGCTTGGAACTGGGTTGTTG | for qRT-PCR |
| --- | --- | --- |
| Q_PAL_R | AGCACCATTCCAACCCTTTA |  |
| Q_C4H_F | TTTGGGTCGTTTGGTACAGA | for qRT-PCR |
| Q_C4H_R | AAAATTGCCTTGGCTTAGCA |  |
| Q_4CL_F | AAGGTGCACTTTGTTCATGC | for qRT-PCR |
| Q_4CL_R | CGTTGCAATTTAAAAGCCAAAT |  |
| Q_CHS_F | CAGGAGAAGGACTGGAGTGG | for qRT-PCR |
| Q_CHS_R | AGCAGCAACACTATGGAGCA |  |
| Q_CHI_F | ATGGAGTTTCTCCTCCAGCA | for qRT-PCR |
| Q_CHI_R | GGTTTTTCACTGTCGACTCCA |  |
| Q_F3H_F | CTGAAGAAGCTGGCCAAAGA | for qRT-PCR |
| Q_F3H_R | TGCAAGGATTTCCTCCAATG |  |
| Q_F3'H_F | GCTGATGTTAGGGGCAATGA | for qRT-PCR |
| Q_F3'H_R | CTCACCATGAAACGACAACG |  |
| Q_F3'5'H_F | AAACATGGATGAGGCCTTTG | for qRT-PCR |
| Q_F3'5'H_R | GCAAGGGATGTGCTTAGGAA |  |
| Q_ANS_F | GCCACCGAAGGATAAGATCA | for qRT-PCR |
| Q_ANS_R | TGGGTCTTCCTGAACAGCTT |  |
| Q_LAR_F | GAATGAGCCATTCCGAACAT | for qRT-PCR |
| Q_LAR_R | GCTTCGACTACTGGCTTTGG |  |
| Q_GSTF12_A_F | ACATCTACGAGCAACGCTTG | for qRT-PCR |
| Q_GSTF12_A_R | ATGTGCCACATCCCAACATC |  |
| Q_GSTF12_D_F | ACATCTACGAGCAACGCTTG | for qRT-PCR |
| Q_GSTF12_D_R | ATGTGCCACATCCCAACATC |  |
| Q_UBQ7_F | GAAGGCATTCCACCTGACCAAC | for qRT-PCR |
| Q_UBQ7_R | CTTGACCTTCTTCTTGTGCTTG |  |
